# Selected Activities of *Citrus Maxima* Merr. Fruits on Human Endothelial Cells: Enhancing Cell Migration and Delaying Cellular Aging

**DOI:** 10.3390/nu6041618

**Published:** 2014-04-21

**Authors:** Paiwan Buachan, Linda Chularojmontri, Suvara K. Wattanapitayakul

**Affiliations:** 1 Department of Pharmacology, Faculty of Medicine, Srinakharinwirot University, Bangkok 10110, Thailand; E-Mail: nohin@hotmail.com; 2 Department of Preclinical Science, Faculty of Medicine, Thammasat University, Pathum Thani 12121, Thailand; E-Mail: ning.phd@gmail.com

**Keywords:** aging, citrus, endothelial cells, HUVEC, pomelo, pummelo, senescence, oxidative stress

## Abstract

Endothelial injury and damage as well as accumulated reactive oxygen species (ROS) in aging play a significant role in the development of cardiovascular disease (CVD). Recent studies show an association of high citrus fruit intake with a lower risk of CVD and stroke but the mechanisms involved are not fully understood. This study investigated the effects of pummelo (*Citrus maxima* Merr. var. Tubtim Siam, CM) fruit extract on human umbilical vein endothelial cell (HUVECs) migration and aging. The freeze-dried powder of fruit extract was characterized for antioxidant capacity (FRAP assay) and certain natural antioxidants, including ascorbic acid, gallic acid, hesperidin, and naringin (HPLC). Short-term (48 h) co-cultivation of HUVECs with CM enhanced cell migration as evaluated by a scratch wound assay and Boyden chamber assay. A long-term treatment with CM for 35 days significantly increased HUVEC proliferation capability as indicated by population doubling level (PDL). CM also delayed the onset of aging phenotype shown by senescence-associated β-galactosidase (SA-β-gal) staining. Furthermore, CM was able to attenuate increased ROS levels in aged cells when determined by 2′,7′-dichlorodihydrofluorescein diacetate (DCDHF) while eNOS mRNA expression was increased but the eNOS protein level was not changed. Thus, further *in vivo* and clinical studies are warranted to support the use of pummelo as a functional fruit for endothelial health and CVD risk reduction.

## 1. Introduction

Citrus fruits are among the most valuable functional diets shown to lower oxidative-related disease risks, particularly cardiovascular disease (CVD) [1,2]. The antioxidant constituents of citrus fruits, such as ascorbic acid and flavonoids, scavenge reactive oxygen species (ROS) and hence prevent ROS accumulation and oxidative damage. In CVD, the endothelium is the main target of ROS-induced tissue injury, leading to endothelial dysfunction and premature aging: the aging process is an independent risk factor for the development of CVD. Importantly, endothelial senescence is associated with endothelial dysfunction and pathology of vascular disease, including hypertension and atherosclerosis [3]. A unified pattern observed in endothelial cells under oxidative stress and aging is the impairment of nitric oxide (NO) production and reduction of NO bioavailability due to oxidative destruction by ROS [4]. In chronic oxidative stress, endothelial damage is an initial insult to the vessel wall, where subsequent phenomena lead to macrophage infiltration, proliferation of smooth muscle cells, and vascular remodeling [5]. The endogenous restoration of endothelial integrity is achieved by the migration and proliferation of endothelial cells to heal the injured site or re-endothelialization by the circulating endothelial progenitor cells (EPCs). Substances that promote endothelial cell migration or enhance regenerative capacity of EPCs may have an important role in endothelial tissue repair, restoration of endothelial function, and reduction of CVD risks.

*Citrus maxima* (Burm.f.) Merr. (Syn.: *C. grandis* Osbeck; *C. decumana* L.) is a tropical fruit called Som-O in Thai and pummelo (pomelo) in English. Several recent studies have demonstrated that the cytoprotective action of citrus fruits is enhanced by the presence of antioxidants including vitamin C, phenolics, carotenoids [6] and flavonoid [7,8]. Additionally, epidemiological studies reveal a strong correlation between high levels of citrus fruit consumption and CVD risk reduction, but the mechanisms of action, particularly on endothelial cells and cardiac cells, have not been fully explored [9,10]. Thus, this study aimed to investigate the effects of pummelo fruit extract on endothelial cell migration, prevention of oxidative stress, and delay of endothelial aging. Human umbilical vein endothelial cells (HUVECs) were used in the experiments for the scratch wound assay and Boyden chamber cell migration assay. HUVECs were maintained in long-term cultures to mimic cellular aging. Senescence characteristics of HUVECs were determined by population doubling level (PDL), senescence-associated (SA) beta-galactosidase staining, and eNOS expression.

## 2. Materials and Methods

### 2.1. Chemicals

Chemicals and reagents used in this study were high quality grade and acquired from Sigma-Aldrich (St. Louis, MO, USA) or otherwise indicated. Cell culture media M199 and supplements were purchased from Invitrogen (Carlsbad, CA, USA). Culturewares and 96-well plates were supplied by Nunc Thermo Scientific (Langenselbold, Germany). Total RNA extraction reagent and polymerase chain reaction (PCR) amplification kits were obtained from Invitrogen (Carlsbad, CA, USA) and Bio-Rad (Hercules, CA, USA), respectively. Antibodies for Western blot analysis were obtained from Santa Cruz Biotechnologies, Inc. (Dallas, TX, USA) and GE Health Care (Pittsburgh, PA, USA).

### 2.2. Preparation of C. maxima (CM) Fruit Extract

CM variety “Tubtim-Siam” (CM) were harvested from Nakhonsithammarat province, Thailand. The peel was removed and the red edible fresh was collected and processed with a fruit juice extractor. The juice was then filtered through No.1 Whatman filter paper (GE Healthcare Life Sciences, Pittsburgh, PA, USA) prior to the freeze-drying process. The freeze-dried sample yielded 10.1% w/v and was kept at 4 °C until needed. The preliminary results showed that CM at concentrations of 10–1000 μg/mL was not toxic to HUVECs using a methylthiazoletetrazolium (MTT) assay for cell viability evaluation. Thus, throughout the experiments in this study, we applied CM at 10–1000 μg/mL.

### 2.3. Determination of Antioxidant Capacity and Total Phenolic Compounds

Antioxidant capacity was determined by Ferric Reducing Antioxidant Power (FRAP) assay as previously described [11]. The antioxidant capacity of the fruit extract powder is presented as a FRAP value (μmole Fe^2+^/L fruit juice). For the determination of total phenolics, the Folin-Ciocalteau (FC) method is performed as described by Singleton *et al.* with some modifications [12]. FC reagent (Sigma F9252, St. Louis, MO, USA) is added to the sample or gallic acid (GA) assay solution in the presence of Na2CO3. Following a 30 min incubation at 40 °C, the reaction mixture is monitored at 756 nm (Shimadsu UV-1601, Kyoto, Japan). The total phenolic content is calculated as GA equivalent (GAE, mg/L fruit juice).

### 2.4. Determination of Ascorbic Ccid, Gallic Ccid and Certain Citrus Flavonoids by HPLC

The amounts of the common fruit antioxidants ascorbic acid, gallic acid as well as the main citrus flavonoids, including hesperidin and naringin, were determined by high performance liquid chromatography (HPLC). Standard curves of each pure agent (Sigma, St. Louis, MO, USA) were generated and the antioxidant contents were determined from their corresponding standard curves. The conditions of HPLC analyses are described in Table 1 [13,14].

### 2.5. Endothelial Scratch Wound and Cell Migration Assays

HUVECs were obtained from trypsin extraction using the method described previously [15]. Briefly, human umbilical cords were collected under a sterile condition from the labor room of the university hospital and processed within 48 h. Cords were washed with phosphate buffer saline (PBS) and enzyme digestion (0.1% collagenase) was performed at 37 °C for 30 min. HUVECs were eluted with sterile PBS and collected by centrifugation at 1500 rpm for 5 min. HUVECs were cultured in M199 supplemented with 20% fetal bovine serum (FBS), antibiotic, and antimycotic agents (Invitrogen, Carlsbad, CA, USA), in a humidified atmosphere of 95% air and 5% CO_2_ at 37 °C.

**Table 1:** Conditions for high performance liquid chromatography (HPLC) analyses of certain antioxidants in *Citrus maxima* (Burm.f.) Merr (CM).

Agent	Mobile Phase	Flow Rate (mL/min)	Detection λ (nm)	Retention Time (min)	Reference
Ascorbic acid	100 mM phosphate buffer (pH 2.5) 95%: methanol 5%	0.4	243	6.1	[13]
Hesperidin and Naringin	12 mmol Heptafluorobutyric Acid in 0.05% Formic acid 80%: acetronitrile 20%	1.2	283	6.8 and 7.3, respectively	[14]
Gallic acid	0.02% dihydrogen phosphate 95%: acetronitrile 5%	1.0	252	4.1	[16]

**Figure 1:**
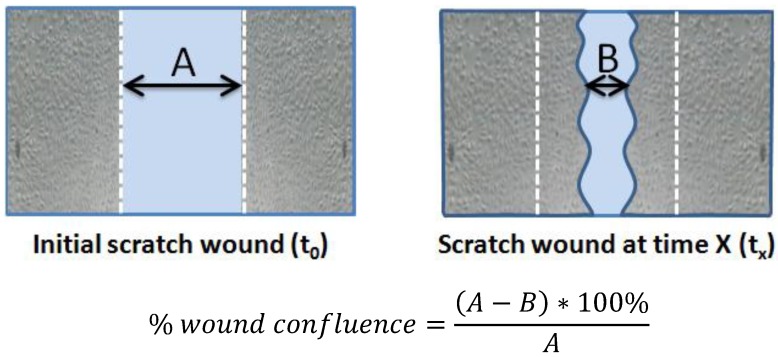
Calculation of percent wound confluence. A: the width of initial scratch wound, B: the width of scratch wound at time 24 or 48 h.

Cell migration was evaluated by the ability to (1) migrate into an empty space created by the *in vitro* scratch wound and (2) migrate through a membrane with an 8-μm pore size, described as a Boyden Chamber assay. The scratch wound assay was performed in low serum (1% FBS) as previously described [15]. For the scratch wound assay, HUVECs were seeded in six-well plates at the density of 5000 cells/cm^2^ and cultured until reaching 100% confluence. The culture medium was then replaced with a medium supplemented with 1% FBS for 24 h. Scratch wounds were created using a sterile 200 μL pipette tip and designated as time 0 h. Photos of the wounds were captured by a digital camera (Olympus DP20, Tokyo, Japan) at the same positions at 0, 24 and 48 h for later calculation (see Figure 1).

In a separate experiment, the Boyden chamber assay was performed to evaluate the chemo-attractive effect of CM using vascular endothelial growth factor (VEGF) (25 ng/mL) as a positive control. HUVECs suspension (2 × 10^5^ cells/mL) was seeded into the upper chamber while a medium vehicle (control group), 25 ng/mL VEGF or CM, were added to the lower chambers. Following a 16 h incubation, the upper chambers were then transferred to a new plate containing 0.25% trypsin-ethylenediaminetetraacetic acid (EDTA) solution and 5 μM calcein-acetoxymethyl ester (CAL-AM, Sigma). Then the plate was further incubated at 37 °C in for 45 min. Migrated cells were evaluated by the levels of fluorescent products generated by the hydrolysis of CAL-AM, which was monitored at 485/528 nm.

### 2.6. Cell Culture and Determination of Population Doubling Level (PDL)

At Passage 3, cells were seeded into a six-well plate at the density of 5 × 10^4^ cells/well. After 4–5 days in culture, cells were harvested by 0.05% trypsin containing 1 mM EDTA. The number of cells was counted by a hemocytometer and recorded for calculation. Cells were reseeded into a six-well plate with the same starting number of 5 × 10^4^ cells and repeated culture until reaching Passage 10. Morphological changes were observed under an inverted microscope (Olympus DP20, Tokyo, Japan). The population doubling level (PDL) was calculated according to the equation: *n* = 3.32 (log UCY − log L) + X, where *n* = the final PDL number at the end of each subculture, UCY = the cell yield at that point, L = the cell number used for starting, and X = the PDL number of the starting subculture [17].

### 2.7. Determination of Intracellular ROS

Intracellular ROS was determined by fluorescent intensity of dichlorodihydrofluorescein (DCF) generated from the interaction of the nonfluorescence 2′,7′-dichlorodihydrofluorescein diacetate (DCDHF) and ROS. The accumulation of ROS in aged (P.10) cells was compared with young cells (P.3). Fluorescent intensity was measured at excitation/emission 485/528 nm using a fluorescence plate reader (BioTek^®^ Synergy HT, Winooski, VT, USA).

### 2.8. Senescence-Associated β-Galactosidase (SA-β-gal) Staining

Cells at Passage 10 were washed twice with PBS and then fixed with 2% formaldehyde and 2% glutaraldehyde in PBS for 5 min. After the PBS washes, cells were incubated with β-galactosidase substrate staining solution (1 mg/mL 5-bromo-4-chloro-3-inolyl-β-*D*-galactoside (X-gal) in dimethyformamide, 40 mM citric acid/sodium phosphate (pH 6.0), 5 mM potassium ferrocyanide, 5 mM potassium ferricyanide, 150 mM NaCl, 2 mM MgCl_2_) at 37 °C without CO_2_ for 8–12 h [18]. Senescent cells were identified as blue-stained cells under the inverted microscope and counted at a minimum of 500 cells to determine the percentage of SA-β-gal-positive cells.

### 2.9. Analyses of eNOS mRNA and Protein Expression

eNOS expression was evaluated in HUVECs at Passage 3 and 15. Based on the preliminary results shown, no change was detected at Passage 10. The eNOS mRNA expression was determined by reverse-transcriptase polymerase chain reaction (RT-PCR). Total RNA was extracted from samples using Trizol reagent (Invitrogen). RNA was converted into cDNA using the iScript cDNA Synthesis kit (Bio-Rad, Hercules, CA, USA). The transcribed cDNA was then used for PCR amplification to estimate the relative expression of eNOS specific to primers (forward, 5′-GACGCTACGAGGAGTGGAAG-3′; reverse, 5′-CCTGTATGCCAGCACAGCTA-3′, product size = 197 bp). PCR amplification was performed using *Taq* DNA polymerase (Invitrogen) for 30 cycles with an annealing temperature of 58 °C. PCR products were then run on 1.8% agarose gel and visualized by ethidium bromide. The images were captured by Genesnap software (Syngene, Cambridge, UK) and analyzed with the ImageJ program (National Institutes of Health, Bethesda, MD, USA).

For Western blot analysis of eNOS protein expression, HUVECs were harvested and lyzed in a buffer containing 20 mM Tris–HCl (pH 7.2), 130 mM NaCl, 1% NP-40, and 1% protease inhibitor cocktail (Sigma-Aldrich, St. Louis, MO, USA). Equal amounts of protein samples were loaded and separated by 10% sodium dodecyl sulfate polyacrylamide gel electrophoresis (SDS–PAGE) under reducing conditions and then transferred to a polyvinylidene fluoride (PVDF) membrane. The relative eNOS expression was determined using the activity of an enhanced chemiluminescence (ECL) detection kit (GE Healthcare Life Sciences, Pittsburgh, PA, USA) against eNOS antibody (Santa Cruz Biotechnology Inc., Dallas, Texas, USA). The relative expression of eNOS was then calculated using the density of β-actin bands as references for ratio expression.

### 2.10. Statistical Analysis

The data were expressed as means ± SEM (*n* ≥ 3) and statistical significance was calculated using one-way or two-way ANOVA with a Bonferroni post-test. Statistical significance was determined at *p* < 0.05.

## 3. Results

### 3.1. Antioxidant Capacity and Certain Antioxidant Compositions in CM Fruit

**Figure 2:**
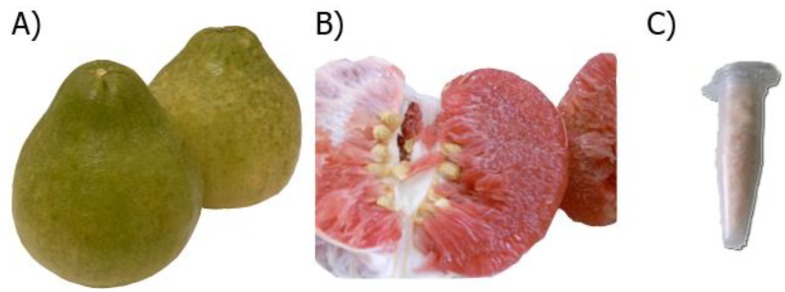
*Citrus maxima* Merr. (pummelo) fruits var. Tubtim-Siam. (**A**) general appearance; (**B**) the reddish inner flesh; (**C**) pinkish freeze-dried powder.

The color of freshly squeezed fruit juice was only slightly pink whereas the red color attribute, known as lycopene, remained with the pulp. The freeze-dried product of CM fruit extract was a pinkish white dry powder representing a 10.1% (w/v) yield (Figure 2). One gram of the dry powder was equivalent to 9.9 mL of fruit juice. Table 2 demonstrates antioxidant power, total phenolics, HPLC analysis of ascorbic acid and certain flavonoids in the fruit juice.

**Table 2:** Antioxidant capacity, total phenolic contents, ascorbic cotent, and certain flavonoids.

Content	FRAP Value (μmol Fe^2+^/L)	GAE (mg/L)	Content in Dry Powder %(w/w)	Content in Fruit Juice (mg/L)
Total antioxidant power	6609			
Total phenolics		690		
Ascorbic acid			0.476	423.5
Gallic acid			0.064	57.0
Hesperidin			0.100	89.1
Naringin			0.542	482.3

### 3.2. CM Enhanced Endothelial Cell Migration

**Figure 3:**
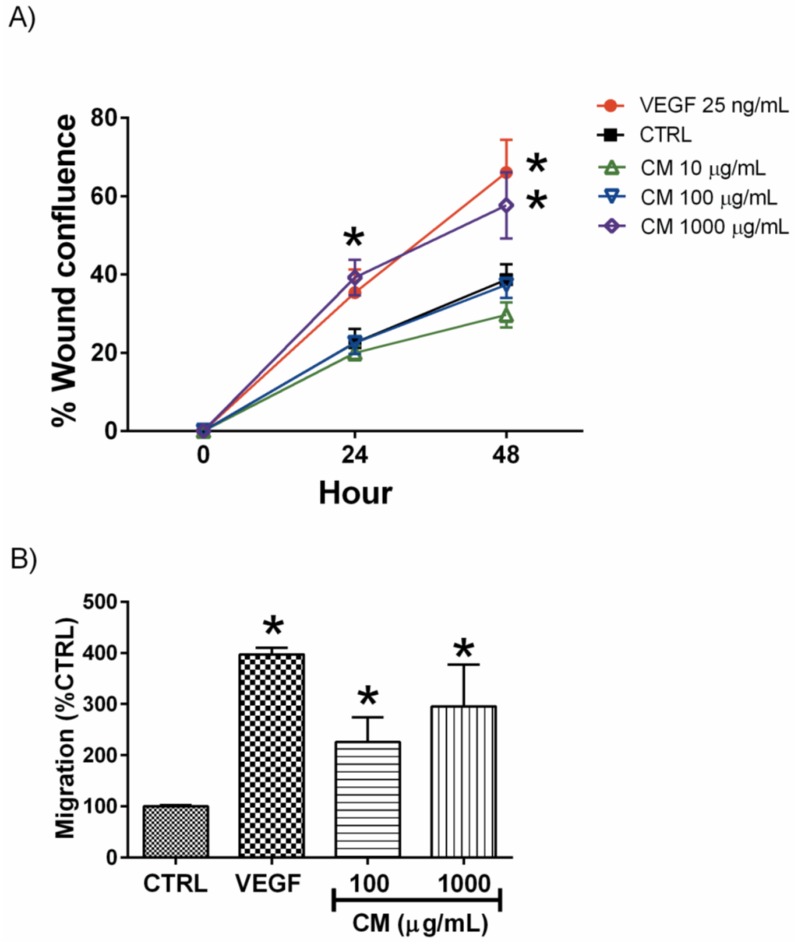
Effects of CM on endothelial wound closure and cell migration. (**A**) Percentage of wound confluence indicating the rate of endothelial wound closure at 24 and 48 h when comparing CM treatment with vehicle treated cells (CTRL); (**B**) Endothelial cell migration using Boyden chamber assay as described in Material and Methods. * *p* < 0.05 *vs.* CTRL.

Scratch wound assay and Boyden Chamber cell migration assays are the most commonly used tools for the evaluation of cell migration. The two assays confirmed that CM enhanced endothelial cell migration. In the scratch wound assay, VEGF (25 ng/mL) significantly increased the wound closure area two-fold compared to that of the vehicle treated control (66.06% ± 8.42 *vs.* 38.67% ± 3.96%, respectively, *p* < 0.05). CM only improved wound confluence by 20% (57.67% ± 8.42%) at the high concentration of 1000 μg/mL, wheras the lower concentration did not alter the wound-healing rate (Figure 3A). On the other hand, CM as low as 100 μg/mL accelerated HEVEC migration by approximately two-fold in the Boyden Chamber assay (Figure 3B).

### 3.3. CM Modified HUVEC Population Doubling Level (PDL)

**Figure 4:**
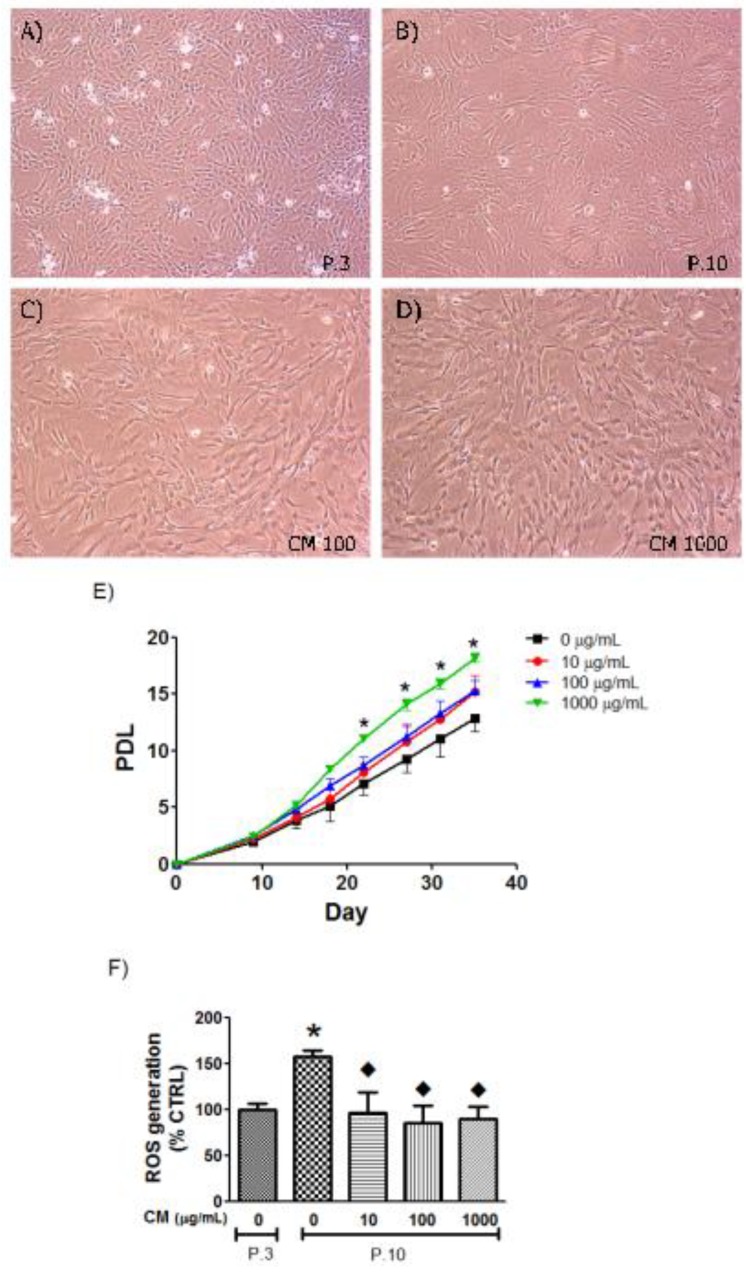
Population doubling level (PDL) of human umbilical vein endothelial cell (HUVECs). Representative photographs of HUVEC morphology at different passages (40× objective lenses): (**A**) HUVECs Passage 3 (P.3); (**B**) HUVECs Passage 10 (P.10); (**C**) HUVECs P.10 with long-term treatment of CM 100 0 μg/mL); (**D**) HUVECs P.10 with long-term treatment of CM 1000 μg/mL); (**E**) Cumulative PDL up to day 35; (**F**) Intracellular reactive oxygen species (ROS) level of HUVECs comparing P.3, P.10 and P.10 with long-term CM co-culture. * *p* < 0.05, P.3 *vs.* P.10 non-treated groups (0 μg/mL); ◆, *p* < 0.05, P.10 non-treated group *vs.* P.10 treated with CM 1000 μg/mL.

HUVECs at young passages were characterized as adherent cells exhibiting a spindle and round shaped cobblestone appearance, but cells at later passages (old cells) progressively changed to a large and flattened shape (Figure 4). During 35 days of cultivation, HUVECs treated with CM at a concentration of 1000 μg/mL significantly increased PDL from Day 22 (Passage 7) when compared with the vehicle treated HUVECs (Figure 4A–D). Additionally, the flattening pattern at Passage 10 was ameliorated and the ability of cells to populate was increased. CM at lower concentrations (10 and 100 μg/mL) slightly modified PDL but did not reach statistical significance (Figure 4E).

### 3.4. CM Decreased Intracellular ROS in Late Passage Cells

Intracellular ROS formation in HUVEC at Passage 10 was increased 1.6-fold, which was significant when compared with the young cells at Passage 3. As illustrated in Figure 4F, the long-term treatment of HUVEC from Passage 3 to Passage 10 with CM at 10, 100, and 1000 μg/mL significantly decreased intracellular ROS generation to the levels that were comparable to the young HUVECs.

### 3.5. SA-β-Gal Activity Decreased with CM Treatment

**Figure 5:**
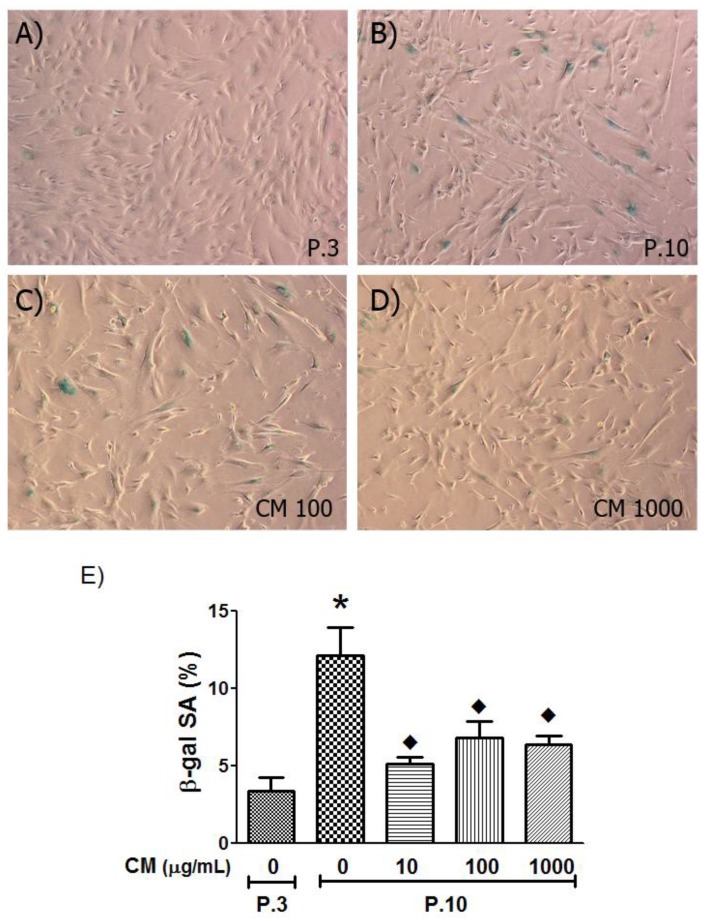
Senescence associated beta-galactosidase (SA-β-gal) staining of HUVECs. (**A**) to (**D**) (40× objective lenses) at Passage 3 (P.3) and Passage 10 (P.10) with or without CM (100, 1000 μg/mL) treatment as indicated in the figures. (**E**) Proportion of SA-β-gal positive staining in HUVECs in young cells (P.3) and aged cells (P.10) with or without CM co-culture. * *p* < 0.05, P.3 *vs.* P.10 non-treated groups (0 μg/mL); ◆, *p* < 0.05, P.10 non-treated group *vs.* P.10 treated with CM 1000 μg/mL.

The senescence levels of HUVECs were investigated using SA-β-gal staining, a widely recognized biomarker of cellular aging. Cells with prolonged *in vitro* cultivation resulted in enhanced levels of SA-β-gal-positive cells (3.6-fold increase) while HUVECs treated with CM at all concentrations significantly decreased senescence cells by 50% on average (Figure 5).

### 3.6. Alteration in eNOS Expression

**Figure 6:**
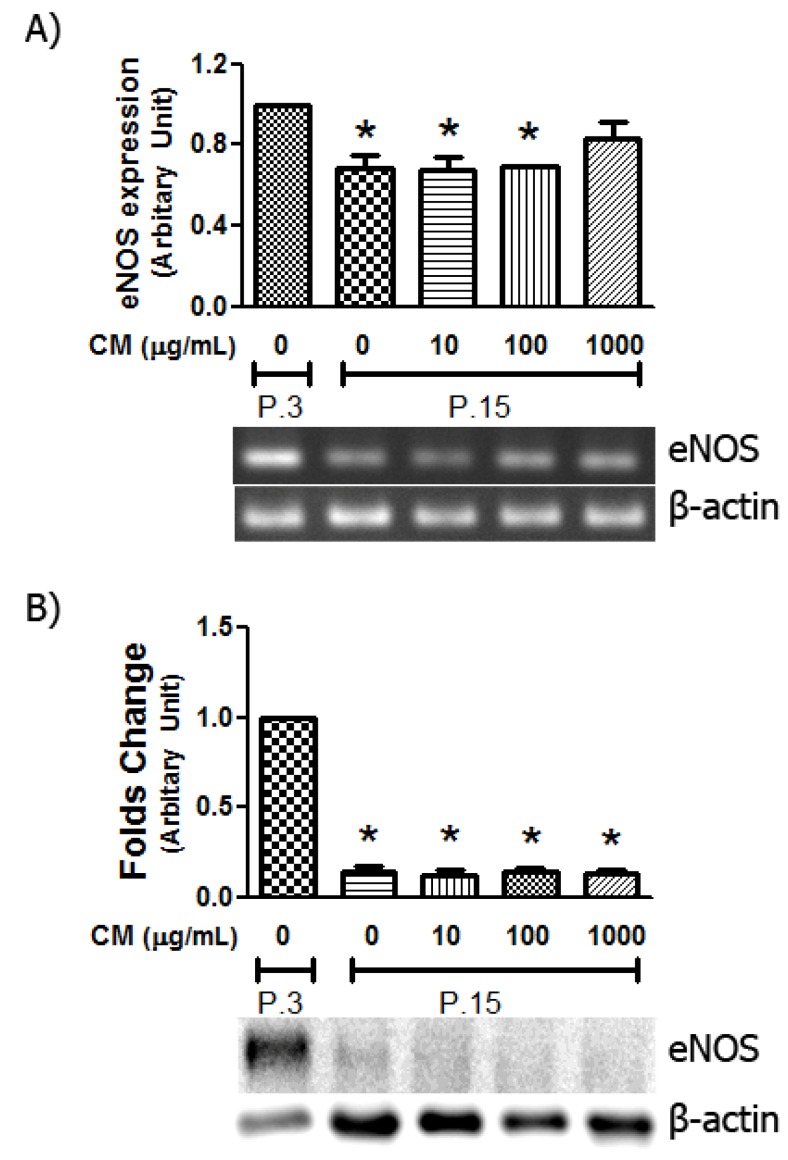
Influence of CM on eNOS expression. The expression of eNOS mRNA and protein were evaluated by reverse transcription-polymerase chain reaction (RT-PCR) and Western blot analysis, respectively. (**A**) Semi-quantitative eNOS mRNA expression was evaluated using specific eNOS primer and normalized to the house keeping gene β-actin as described in Materials and Methods. (**B**) The relative eNOS protein expression in HUVECs at late passage was compared to passage 3 (P.3). * *p* < 0.05 *vs.* P.3.

Figure 6 shows that eNOS mRNA expression was significantly down regulated in HUVECs in aged cells at late passage. When applied at low CM concentrations (10 and 100 μg/mL), no change in eNOS expression was observed in HUVEC. However, CM at 1000 μg/mL increased mRNA expression to the level that was not significantly different from HUVECs at the young passage (Figure 6A). For eNOS expression at the protein level, Western blot analysis revealed that the amount of eNOS protein detected in the late passage of HUVECs was suppressed to 13% (Figure 6B). Long-term CM co-cultivation at all concentrations did not alter the presence of eNOS protein in HUVECs, although a change in mRNA was observed in HUVECs treated with CM 1000 μg/mL.

## 4. Discussion

Endothelial dysfunction and aging are recognized as major contributors to the development and progression of atherosclerosis and other CVD. The ability of endothelial cells to recover and heal the damage is important in tissue repair, which inversely correlates to CVD risk. Strategies to prevent or impede the progression of CVD are not only to promote endothelial health to repair the damage, but also to delay the aging process of cardiovascular tissue [19]. Recent epidemiologic studies have shown the role of natural antioxidants from citrus fruits in lowering CVD risk and stroke, but the mechanisms of action are not fully understood [2,20]. Here we found that the tropical citrus fruit pummelo (*C. maxima* Merr., var. Tubtim Siam, CM) enhanced endothelial wound closure and cell migration, which are an important properties of endothelial cells in order to be resilient to tissue injury. Additionally, when applying CM to long-term HUVEC culture, it was effective in reducing ROS accumulation and delaying cellular aging characters and phenotype. Although CM enhanced eNOS mRNA expression, the protein level was not altered in aged cells. These findings support the consumption of pummelo as one of functional fruits for cardiovascular health.

Two major processes account for endothelial wound repair, cell migration and cell proliferation. Cell migration is the primary event to heal the wound, and when this function is impaired, the wound healing process is hindered in spite of the intact proliferation process [21]. In this study, CM-enhanced endothelial wound closure was likely to stem from cell migration since no significant cell proliferation was observed under low serum conditions (MTT assay, data not shown). Mechanical endothelial damage activates cell migration through the extracellular signal-regulated kinase (ERK) pathway, which is mediated by the fibroblast growth factor-2 (FGF-2) [22] whereas the main regulators for endothelial migration in angiogenesis are VEGF and other angiogenic factors [23].

Since it has been recognized that a long-term accumulation of ROS or chronic oxidative stress contributes to vascular endothelial dysfunction and aging, a myriad of studies have been focusing on the use of antioxidants to benefit the cardiovascular system. Nonetheless, there are conflicting results about the efficacy of antioxidant vitamin supplements, and a large meta-analyses recently concluded that there is no benefit to taking these synthetic vitamin supplements to prevent CVD [24]. On the contrary, epidemiological studies consistently show advantages of high fruit and vegetable consumption in lowering the risks of CVD and stroke. Among fruits and vegetables, citrus fruits are well evidenced in their potential use as dietary supplements to combat CVD and stroke [2,25,26,27]. These long-term cohort studies are in agreement with our continuing co-cultivation of CM with endothelial cells up to 35 days. CM appears to delay replicative senescence and reduce ROS accumulation, which has been implicated in aging endothelial cells. This vascular aging phenomenon is associated with an impairment of redox signaling and telomere integrity, which results in dysregulation of vascular homeostasis [19,28].

Cellular senescence is a key determinant of cells entering the aging process. Several morphological and biochemical changes are detected in aging cells. Aging phenotype is represented by increased cell size, flat shape, increased granulation, enhanced positive SA-β-gal staining, and others. In endothelial cells, changes in morphology, cellular function and gene expression are well described, such as decreased NO production, alterations in cyclin-dependent kinase, p53, and p66^Shc^ [29]. SA-β-gal is the most widely used biomarker of aging cells. HUVECs at later passages have increased SA-β-gal-positive cells and are proportionate to passage number [30]. In our study, prolonged cultivation of HUVECs with the peculiar indigenous citrus fruit delayed the aging phenotype. Additionally, CM significantly increased cell proliferation ability as measured by PDL, which refers to the total number of times the cells in the population have doubled since their primary isolation *in vitro* [31]. CM-enhanced cell proliferation is associated with the survival of cell populations that reflect the maintenance of endothelial health and function. However, the benefits of CM on endothelial aging are limited to ROS, morphology, and the ability to grow; however, it did not change the eNOS protein level, despite the increasing mRNA level.

The synthesis of NO via eNOS is one of the crucial functions of endothelial cells. The regulation of eNOS expression occurs at multiple stages, including modifications at post-transcription, post-translation, and epigenetic levels. No direct translational proportion of the eNOS mRNA upregulation to eNOS protein expression was observed in long-term CM (1000 μg/mL) treatment. This phenomenon can be explained partly by the alterations of eNOS regulators in aged cells. For example, the silencing information regulator (SIRT1), a NAD-dependent histone deacetylase that is associated with transcriptional silencing, genome stability, and longevity, is down-regulated in aged cells and atherosclerotic vessels *in vivo* [32]. The absence of SIRT1 increases the expression of p66^Shc^ and promotes hyperglycemia-induced endothelial dysfunction [33]. On the other hand, a substance that activated endothelial SIRT1 expression protected cells against H_2_O_2_ insult and delayed endothelial aging phenotype [34]. Thus, a substance that modifies SIRT1 may benefit the eNOS expression in aged cells, although this needs further investigation.

The advantage of consuming high antioxidant diets over vitamin supplements is presumed to be the consequences of bioactive compounds, such as flavonoids, acting on multi-cellular targets rather than chemical scavenging properties. The flavonoids almost exclusively found in citrus fruits are “citrus flavanones”, such as aglycones (hesperetin, naringenin, eriodictyol, and others) and their glycosides (hesperidin/neohesperidin, naringin/narirutin, eriocitrin/neoeriocitrin, respectively) [35]. The antioxidant activities of these flavanones are well recognized and their pharmacological benefits on the cardiovascular system have been demonstrated both in the laboratory and clinical studies. For instance, hesperidin prevents human endothelial inflammation by multiple mechanisms, including suppressing expression of vascular cell adhesion molecule-1 (VCAM-1), modifying hypoxia inducible factor 1-alpha (HIF-1α), and inhibiting inflammatory cytokine production such as interleukin (IL)-1beta, IL-8, and tumor necrosis factor-alpha (TNF-α) [36,37]. Administering hesperidin orally (500 mg/day, 3 weeks) in human subjects significantly improves endothelial function and reduces circulating biomarkers of inflammation through the mechanisms of enhanced NO production and the suppression of inflammatory mediators such as VCAM-1 and TNF-α [38]. Similarly, naringin, the predominant citrus flavanone found in grapefruit (approximately six-fold higher than hesperidin), reduces risk factors in developing atherosclerosis by inhibiting plague progression and expression of genes related to leukocyte adhesion to the endothelium in a mouse model on a high fat diet [39]. A clinical trial conducted in hypercholesterolemic subjects demonstrates that naringin intake (400 mg/day, 8 weeks) lowered total plasma cholesterol and LDL by 14% and 17%, respectively, while no change in triglyceride was observed [40]. Some studies show conflicting data related to the effects of hesperidin or naringin on cardiovascular-related parameters, which can be explained partially by the phramacokinetics of the citrus flavanones. Hesperidin and naringin are poorly absorbed in the gastrointestinal tract, thereby comparing their effects is complicated if flavonoid blood concentrations are not achieved. Another antioxidant constituent commonly found in citrus fruits is ascorbic acid, which may be taken into consideration for the CM effect. An *in vivo* study shows that vitamin C minimizes oxidative stress and endothelial inflammation in type 1 diabetes [41]. It also increases NO bioavailability by various mechanisms involving BH_4_ and eNOS [42]. Thus, it is possible that the vitamin C content of pummelo may also contribute to the effect of the extract on endothelial function. A meta-analysis of flavanones in grapefruit shows that on average, grapefruit contains 17 ± 9.6 mg naringin, 3 ± 3.4 mg hesperidin, and 5 ± 3.4 mg narirutin per 100 g fruit or fruit juice, respectively [35]. Red and pink grapefruit contain lower amounts of flavanones than white grapefruit; however, CM var. Tubtim-Siam used in this study consists of 284% and 297% and hence higher concentrations than average for the amounts of naringin (48.23 mg/100 mL) and hesperidine (8.91 mg/100 mL), respectively. While hesperidin is not commonly found in white pummelo, naringin is the major flavanones detected in the range of 24.3–38.7 mg/100 mL [43]. Therefore, Tubtim-Siam pummelo is a great source of citrus flavanones that may potentially be promoted as functional fruit for cardiovascular health.

## 5. Conclusions

The association of endothelial damage and aging in CVD is widely recognized as well as the importance of oxidative stress and its potential therapeutic implications in humans [44]. High consumption of antioxidant-containing fruits and vegetables is a mechanism-based strategy to delay the onset or progression of CVD while regular intake of vitamin supplements fails to prevent or lower CVD risk [45]. Among “cardiovascular fruits”, citrus fruits are well described, and there is evidence in this study that the peculiar variety “Tubtim Siam” contains high levels of bioactive flavonoids. HUVECs treated with the fruit extract improved cell migration and hindered the onset of phenotypical aging. However, the evidence of beneficial effects of this citrus fruit on endothelial cells warrants further animal and human studies before it can be promoted as functional fruit for CVD risk reduction.
